# 1-Kestose as a Candidate Precision Bioactive Component: From GH32-Dependent Gut Microbiota Regulation to Human Health Enhancement

**DOI:** 10.3390/foods15152631

**Published:** 2026-07-27

**Authors:** Tadashi Fujii, Hideaki Takahashi, Eizaburo Ohno, Yoshiki Hirooka, Takumi Tochio

**Affiliations:** 1 Department of Gastroenterology and Hepatology, School of Medicine, Fujita Health University, Toyoake 470-1192, Japan; hideaki.takahashi.xk@fujita-hu.ac.jp (H.T.); eizaburo.ono@fujita-hu.ac.jp (E.O.); t-tochio@fujita-hu.ac.jp (T.T.); 2 Department of Medical Research on Prebiotics and Probiotics, Fujita Health University, Toyoake 470-1192, Japan; yoshiki.hirooka@fujita-hu.ac.jp; 3 BIOSIS Lab. Co., Ltd., Toyoake 470-1192, Japan; 4 Department of Gastroenterology and Hepatology, Fujita Health University Okazaki Medical Center, Okazaki 444-0829, Japan

**Keywords:** 1-kestose, fructooligosaccharides, prebiotic, gut microbiota, glycoside hydrolase family 32 (GH32), inulin, short-chain fatty acids, cross-feeding, human intervention, precision prebiotic

## Abstract

Gut microbiota dysbiosis is implicated in diverse intestinal and systemic disorders, and prebiotics offer a practical strategy to modify host–microbe interactions. This review evaluates 1-kestose as a candidate precision bioactive component by integrating its chemical structure, enzymatic production, gastrointestinal fate, GH32-dependent microbial utilization, human evidence, and qPCR-based response monitoring. Many commercial fructooligosaccharides contain molecules with different degrees of polymerization, complicating structure–function interpretation. In contrast, 1-kestose is a high-purity trisaccharide fructooligosaccharide and the shortest member of the inulin-type fructans. By comparing 1-kestose with long-chain inulin, we examine how fructan chain length may influence colonic fermentation kinetics, substrate availability, and tolerability. We then discuss the role of GH32 substrate specificity in the selective microbial utilization of 1-kestose and related fructooligosaccharides, particularly by bifidobacteria and representative butyrate producers. Next, we review the mechanistic rationale and preclinical evidence for co-administration of 1-kestose and long-chain inulin. Human intervention studies have evaluated 1-kestose across gastrointestinal, metabolic, immune-related, neonatal, oncological, and bowel-habit contexts, with emerging evidence of potential benefits. One healthy-adult trial has also evaluated co-administration with long-chain inulin, although direct comparative trials remain an important future priority. Finally, we propose a research framework that integrates high-purity 1-kestose, GH32-dependent microbial selectivity, and qPCR-based baseline stratification and response monitoring. Prospective, independently replicated trials are needed to establish clinical effectiveness and determine the value of biomarker-guided intervention and combination strategies.

## 1. Introduction

The gut microbiota is increasingly recognized as a key determinant of host physiology, and dysbiosis has been linked to a broad spectrum of diseases, including gastrointestinal, metabolic, immune, and neuropsychiatric disorders [1]. Microbial fermentation of dietary substrates produces short-chain fatty acids (SCFAs), including acetate, propionate, and butyrate, which regulate intestinal barrier function, immune homeostasis, and systemic metabolism. Butyrate, in particular, serves as an important energy source for colonocytes and supports epithelial barrier integrity, while SCFAs more broadly contribute to immune regulation, energy homeostasis, and metabolic-inflammatory signaling [2,3]. Accordingly, microbiota-targeted nutritional strategies are being explored to restore functional resilience of the host–microbe ecosystem.

Prebiotics are defined as substrates that are selectively utilized by host microorganisms and confer a health benefit [4]. Fructooligosaccharides (FOSs) are short-chain members of the inulin-type fructan family, whereas inulin comprises longer-chain fructans. 1-Kestose (GF2) is a trisaccharide FOS with a DP of 3 and is the shortest member of this family. Unlike many commercial FOS preparations, which contain mixtures of molecules with different DPs, 1-kestose can be prepared as a high-purity single component. This compositional simplicity enables clearer investigation of the relationships among molecular structure, selective microbial utilization, microbial cross-feeding, and clinical outcomes.

Given its structural simplicity and availability as a high-purity single component, this review evaluates 1-kestose as a candidate precision bioactive component and a model for precision prebiotic research. Here, “precision bioactive component” refers to a chemically defined food-derived molecule for which microbial utilization can be mechanistically characterized and individual responses can potentially be estimated and monitored using quantitative microbial markers. We first summarize the chemical structure, enzymatic production, and gastrointestinal fate of 1-kestose, using long-chain inulin as a comparator to examine how fructan chain length may influence colonic fermentation kinetics, substrate availability, and tolerability. We then discuss how the substrate specificity of GH32 enzymes shapes the selective microbial utilization of 1-kestose, with particular attention to bifidobacteria and representative butyrate producers. Next, we integrate these fermentation and microbial-utilization features to explain the rationale for co-administration with long-chain inulin and review the available preclinical evidence. We then examine human intervention studies across gastrointestinal, metabolic, immune-related, neonatal, oncological, and bowel-habit settings. Finally, we discuss future research priorities, including the potential use of qPCR-based microbial markers for baseline stratification and response monitoring, as well as emerging research directions involving the gut–brain axis and animal-health contexts.

## 2. Structure and Chemical Properties of 1-Kestose

### 2.1. Chemical Structure

1-Kestose is both an oligosaccharide and, more specifically, a trisaccharide fructooligosaccharide with a degree of polymerization (DP) of 3. It contains a sucrose core (Glcα1-2βFru) with one additional fructosyl residue attached to the fructose moiety through a β(2 → 1) glycosidic bond. Thus, it comprises one glucose and two fructose residues and represents the shortest member of the inulin-type fructans. Its molecular mass is approximately 504.4 Da. In contrast, inulin comprises longer β(2 → 1)-linked fructan chains, often with DP > 10.

### 2.2. Enzymatic Production and Purification

1-Kestose can be produced from sucrose by transfructosylation using fungal β-fructofuranosidases or fructosyltransferases. *Aspergillus*-derived enzymes have been extensively studied and used for fructooligosaccharide production. For example, a β-fructofuranosidase from *Aspergillus niger* ATCC 20611 converts concentrated sucrose into inulin-type fructooligosaccharides, including 1-kestose, whereas enzymes from other *Aspergillus* species differ in their product specificity and 1-kestose yield [5,6]. During transfructosylation, the enzyme transfers a fructosyl residue from a donor sucrose molecule to the fructose moiety of an acceptor sucrose molecule through a β(2 → 1) linkage. The primary reaction can be represented as follows: 2 sucrose molecules → 1-kestose + glucose.

The enzymatic reaction does not generally produce 1-kestose as the only product. The resulting syrup may also contain residual sucrose, glucose, and higher-DP fructooligosaccharides such as nystose (DP = 4). High-purity 1-kestose therefore requires downstream separation. In a publicly described production process, the 1-kestose-containing syrup is fractionated by chromatographic separation, concentrated, and crystallized. The process describes purification of the 1-kestose fraction to at least 80% before crystallization and recovery of crystalline 1-kestose with a purity of at least 95% [7]. Thus, the availability of high-purity 1-kestose depends on both selective enzymatic synthesis and subsequent fractionation and crystallization.

### 2.3. Resistance to Digestion and Colonic Fermentation

**Figure 1:**
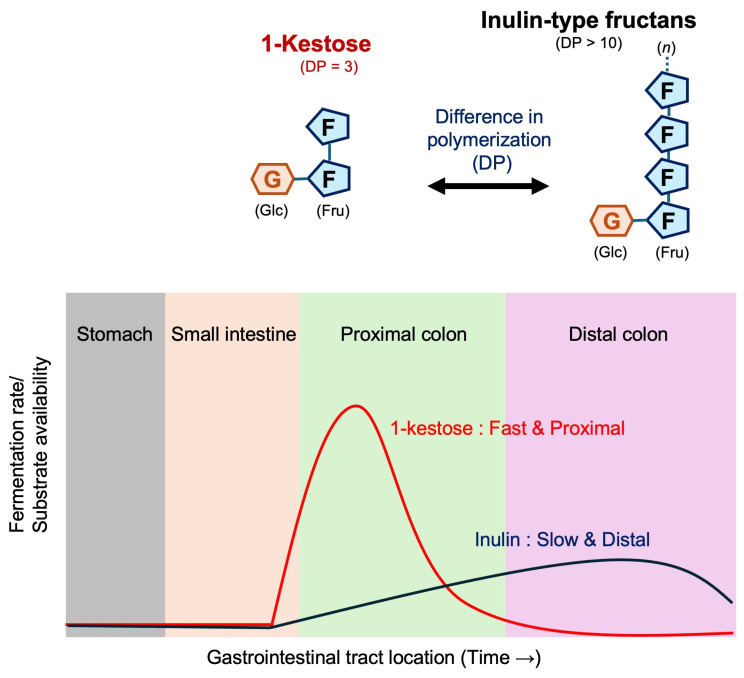
Schematic structures of 1-kestose and long-chain inulin and their conceptual fermentation profiles along the gastrointestinal tract. Low-DP 1-kestose is expected to be fermented more rapidly, whereas long-chain inulin may be fermented more gradually and extend fermentable substrate availability toward the distal colon. The curves illustrate a chain-length-based hypothesis and are not direct in vivo measurements of the specific preparations. Shaded regions indicate the stomach, small intestine, proximal colon, and distal colon.

1-Kestose is a non-digestible carbohydrate that resists digestion by human digestive enzymes and reaches the large intestine with minimal degradation. In the colon, it is fermented by gut microorganisms. As a low-DP fructan, 1-kestose is generally fermented rapidly, particularly in the proximal colon. By comparison, long-chain inulin-type fructans are degraded more gradually and may maintain fermentable substrate availability farther toward the distal colon [8]. Thus, fructan chain length can influence both the rate and spatial distribution of colonic fermentation (Figure 1).

Consistent with these differences, recent human data comparing fructooligosaccharides with inulin indicate distinct microbiota and metabolic responses [9]. Rapid colonic fermentation may contribute to gas production and bloating in susceptible individuals, although tolerability also depends on dose, host characteristics, and background diet. An in vitro study using fibers with different degradation rates—not specifically 1-kestose plus long-chain inulin—reported lower gas production while retaining bifidogenic effects [10]. These observations provide a general rationale for testing whether co-administration of 1-kestose and long-chain inulin can influence colonic substrate availability and tolerability; the available experimental evidence is discussed in Section 4. Interindividual responses to inulin-type fructans also vary with habitual dietary fiber intake and baseline microbiota composition, supporting the value of microbiota-aware stratification in future studies [11,12].

## 3. Microbial Utilization and Microbiota Selectivity via GH32

**Figure 2:**
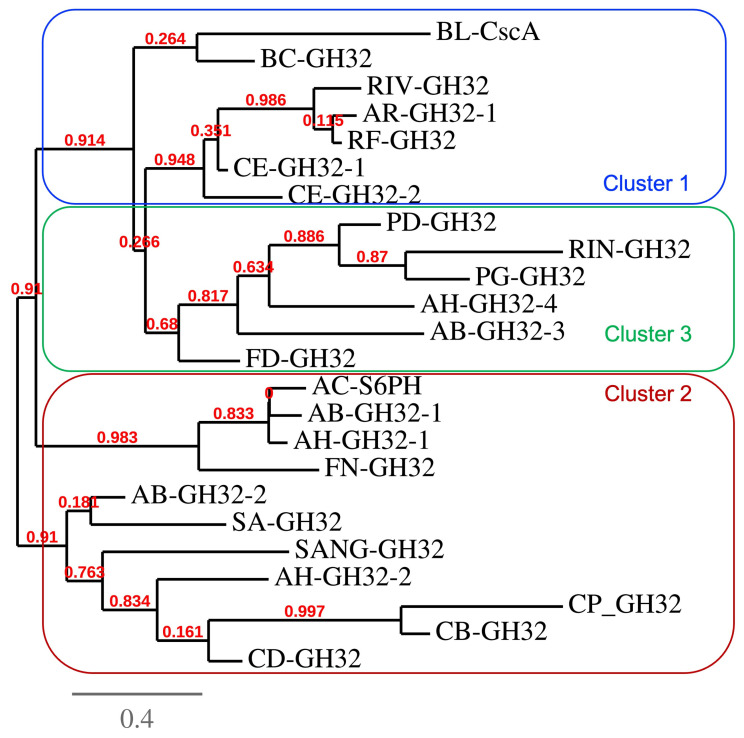
Sequence-based phylogeny and functional clustering of bacterial GH32 proteins. Cluster 1 is associated with 1-kestose and related short-chain fructooligosaccharides, Cluster 2 with a restricted substrate range mainly involving sucrose, and Cluster 3 with longer-chain fructans [13]. Compact protein codes are used in the tree for readability; full organism names, strain information, protein designations, accession numbers, phylogenetic cluster assignments, and supporting references are provided in Supplementary Table S1. The characterized *Parabacteroides* proteins and the characterized *Blautia caecimuris* protein are described in [14,15], whereas proteins from pathogen-associated, disease-associated, and comparator strains are sequence-based predictions supported by [16,17,18,19,20,21]. Cluster assignment alone does not establish substrate specificity. Phylogenetic reconstruction of the GH32 amino acid sequences was performed using the One Click mode of the Phylogeny.fr web platform using default settings. Red numbers at the internal nodes indicate SH-like approximate likelihood-ratio test support values expressed as proportions. The scale bar indicates the estimated number of amino acid substitutions per site.

Glycoside hydrolase family 32 (GH32) enzymes provide the enzymatic basis for microbial degradation of sucrose and fructans of different chain lengths, including 1-kestose, nystose, and longer-chain fructans such as inulin. However, GH32 is a functionally diverse enzyme family, and individual enzymes can differ substantially in their substrate specificity. Previous work showed that bacterial GH32 amino acid sequences can be grouped by phylogenetic analysis into clusters associated with distinct fructooligosaccharide-degrading profiles [13]. Figure 2 applies this framework to compare GH32 enzymes relevant to the utilization of 1-kestose and other fructans.

Cluster 1 contains enzymes associated with low-DP substrates such as 1-kestose and other short-chain fructooligosaccharides, whereas Cluster 3 contains enzymes associated with longer-chain fructans such as inulin. Cluster 2 contains enzymes inferred to have a narrower substrate range, mainly sucrose hydrolysis [13]. These sequence-based clusters suggest differences in substrate specificity among GH32 proteins, but the substrate specificity of individual proteins requires biochemical validation. The following subsections describe intracellular uptake and degradation, extracellular degradation and cross-feeding, and the interpretation of sequence-predicted GH32 proteins.

### 3.1. Intracellular Uptake and GH32-Mediated Degradation

We focused on commensal butyrate producers with characterized GH32 repertoires or fructan-utilization phenotypes, including *Faecalibacterium prausnitzii* and species of *Anaerostipes*, *Roseburia*, and *Eubacterium*. *Bifidobacterium* spp. (e.g., *B. longum* subsp. *longum*, *B. adolescentis*, and *B. pseudocatenulatum*), together with representative commensal butyrate producers such as *F. prausnitzii*, can import intact 1-kestose through oligosaccharide transport systems, often mediated by ABC transporters, and hydrolyze it intracellularly using GH32 [13,22]. Intracellular degradation may limit the release of monosaccharides into the lumen and reduce access by competing microbes. This mechanism is consistent with the bifidogenic responses and acetate production reported after 1-kestose intake. However, transporter activity, GH32 expression, and growth on 1-kestose should be verified for individual strains.

### 3.2. Extracellular GH32-Mediated Degradation and Cross-Feeding

Several representative commensal butyrate producers, including *Anaerostipes hadrus*, *Anaerostipes butyraticus*, *Roseburia intestinalis*, and *Eubacterium rectale*, encode multiple GH32 proteins. Their GH32 repertoires include proteins predicted to be secreted or cell-surface-associated and to degrade long-chain fructans extracellularly [13]. Because long-chain inulin is fermented more gradually than 1-kestose, fermentable substrate may remain available farther along the colon, where extracellular fructan degradation may continue.

Extracellular degradation of long-chain fructans can release shorter fructan fragments that become available to secondary consumers [23]. In parallel, acetate and lactate produced by bifidobacteria can serve as substrates for selected butyrate producers and thereby support butyrate production [24,25]. In the context of 1-kestose, this metabolite-based pathway provides a plausible link between a bifidogenic response and downstream butyrate production. These interactions represent two complementary forms of microbial cross-feeding: the sharing of carbohydrate fragments generated by extracellular degradation and the exchange of fermentation metabolites between bacterial groups. Their implications for the co-administration of 1-kestose and long-chain inulin are discussed in Section 4.

### 3.3. Interpretation of Predicted GH32 Proteins in Pathogen-Associated and Probiotic Comparator Strains

From a safety perspective, it is important to determine whether pathogen- or disease-associated bacteria can directly utilize 1-kestose. Figure 2 therefore compares predicted GH32 proteins from beneficial commensals and pathogen-associated organisms, together with *Clostridium butyricum* CBM588 as a probiotic clostridial comparator strain. The term probiotic is used here according to the consensus definition of live microorganisms that, when administered in adequate amounts, confer a health benefit on the host [26].

The genomes examined for the pathogen- or disease-associated bacteria listed in Supplementary Table S1—*Clostridium perfringens* [16], *Clostridioides difficile* [17], *Staphylococcus aureus* [18], *Fusobacterium nucleatum* [19], and *Streptococcus anginosus* [20]—each encode, at most, one predicted GH32 protein. All of these proteins fall within Cluster 2 in Figure 2. This placement is more consistent with a restricted, sucrose-oriented substrate range than with efficient 1-kestose hydrolysis.

For comparison, the predicted GH32 protein encoded by the probiotic strain *Clostridium butyricum* CBM588 [21] also falls within Cluster 2. Its co-administration with 1-kestose may therefore be more appropriately investigated as a complementary synbiotic approach than as direct stimulation of CBM588 by 1-kestose.

However, sequence-based cluster placement alone does not establish the substrate specificity of these proteins or the ability of the corresponding strains to utilize 1-kestose. Biochemical characterization of the GH32 proteins and growth assays using 1-kestose are needed before direct utilization can be confirmed or excluded.

## 4. Preclinical Evidence for Co-Administration with Long-Chain Inulin

1-Kestose and long-chain inulin differ in both their colonic fermentation kinetics and microbial utilization pathways. 1-Kestose can be rapidly taken up and fermented by bifidobacteria, generating acetate and lactate that may support butyrate production. In contrast, long-chain inulin can be degraded extracellularly by representative butyrate producers and may remain available farther along the colon. These complementary temporal, spatial, and microbial features provide a mechanistic rationale for directly testing their co-administration.

Two ovalbumin-induced mouse studies examined 1-kestose and long-chain inulin individually and in combination in complementary settings: one evaluated the prevention of allergy development, whereas the other investigated the amelioration of established allergic responses [14,27]. In both models, the combined intervention produced stronger or distinct effects on selected allergy-related outcomes, gut microbiota profiles, and acetate production compared with the individual fructans. These findings support further clinical evaluation of the combined approach. Human trials comparing each component alone with their combination are needed to determine whether co-administration provides additional or synergistic benefits.

## 5. Evidence from Human Intervention Studies

Human intervention studies have evaluated 1-kestose across a range of clinical settings, including gastrointestinal, metabolic, immune-related, neonatal, oncological, and bowel-habit contexts. Most studies have examined 1-kestose as a single component and have reported changes in clinical outcomes, gut microbiota composition, and microbial functional markers. One randomized trial in healthy adults additionally evaluated its co-administration with long-chain inulin. Table 1 summarizes the representative human studies discussed in this section.

### 5.1. Inflammatory Bowel Disease

In a randomized, double-blind, placebo-controlled pilot study in patients with mild to moderate ulcerative colitis, 1-kestose supplementation (10 g/day for 8 weeks) increased the clinical remission rate and reduced disease activity, as assessed by the Lichtiger index [28], accompanied by a reduction in the abundance of *Ruminococcus gnavus*, a species often associated with mucin degradation and intestinal inflammation [37,38]. In Crohn’s disease, a pilot study reported that remission remained stable and fecal *nan* gene levels, a mucin-degradation-associated functional marker, decreased during 1-kestose intake (3 g/day for 12 weeks) [29]. While encouraging, these findings require confirmation in adequately powered randomized trials with standardized endpoints and microbiome-aware stratification.

### 5.2. Metabolic Outcomes and Insulin Resistance

A placebo-controlled human trial in adults with overweight demonstrated that 1-kestose supplementation (10 g/day for 12 weeks) significantly reduced fasting serum insulin and improved indices of insulin resistance, alongside an increase in fecal *Bifidobacterium* abundance [30]. Subgroup analyses suggested that baseline metabolic status and host factors (e.g., age) may influence responsiveness, highlighting the importance of responder stratification in future studies.

### 5.3. Pediatric Atopic Dermatitis

In infants with atopic dermatitis (AD), 1-kestose intake was reported to improve SCORAD scores [31]. Because infancy is a critical window for microbiota development, microbiota-targeted interventions may shape immune phenotypes and disease trajectories. The AD study also suggested that 1-kestose supplementation may be linked to establishment of the major butyrate producer *F. prausnitzii*, underscoring the need to interpret clinical outcomes together with microbial and metabolic shifts [31].

### 5.4. Food Allergy

In children with cow’s milk allergy, 1-kestose administration was associated with reduced serum IgE levels and improved clinical tolerance indices, with a potential link between IgE-related measures and the abundance of *Fusicatenibacter* [32], a genus reported to be associated with inflammatory states [39]. Given the multiple confounders in allergy management (topical therapy, dietary restrictions, seasonality, and other interventions), rigorously controlled trials that integrate clinical thresholds with immune markers are needed.

### 5.5. Neonatal Care and NICU Settings

A pilot randomized trial in preterm or low-birth-weight neonates in a neonatal intensive care unit compared *Bifidobacterium breve* M16-V given with either 1-kestose or lactulose once daily for four weeks from birth [33]. Both groups showed increases in microbial diversity over time, while qPCR-based analyses suggested a higher total bacterial load and a tendency toward lower *Staphylococcus aureus* levels in the 1-kestose group [33]. These results suggest that 1-kestose may serve as an alternative prebiotic component for synbiotic regimens in vulnerable neonatal populations.

### 5.6. Supportive Care in Pancreatic Cancer

In a single-center randomized controlled pilot trial in patients with pancreatic ductal adenocarcinoma undergoing chemotherapy, the 1-kestose group received 9 g/day for 12 weeks [34]. The intervention was associated with a significant decrease in the tumor marker CA19-9 and a reduction in the neutrophil-to-lymphocyte ratio, alongside mitigation of declines in albumin and increases in C-reactive protein. Changes in gut microbiota composition, including a reduction in Escherichia coli, were also observed [34]. Given the pilot design and concurrent chemotherapy, these findings should be interpreted cautiously and warrant confirmation in larger trials of microbiota-targeted supportive care.

### 5.7. Bowel Habits and Functional Constipation

In children with constipation, 1-kestose intake (3 g/day for 8 weeks) increased defecation frequency and improved stool form [35]. These findings suggest that 1-kestose may support bowel regulation in pediatric settings.

### 5.8. Healthy Adults and Combined Fructan Intervention

A randomized, double-blind, placebo-controlled trial in healthy adults evaluated combined intake of 1-kestose and long-chain inulin (3 g/day each for 4 weeks) [36]. The combination increased *Bifidobacterium* abundance relative to placebo, indicating that co-administration was feasible under the study conditions and produced a measurable microbiota response. However, because the trial did not include 1-kestose-only or inulin-only groups, it could not determine whether the combination provided effects beyond either component alone or whether the observed effects were additive or synergistic. The preclinical allergy studies provide a rationale for such comparative testing but cannot substitute for direct evaluation in humans. A four-arm factorial trial including placebo, 1-kestose alone, long-chain inulin alone, and the combination, with prespecified interaction analyses, is needed to address these questions.

Taken together, the available human studies provide encouraging evidence for the potential effects of 1-kestose across several clinical settings. However, many studies have been small-scale or exploratory, and study populations, intervention doses, durations, and outcome measures vary. Independent replication also remains limited. Larger, adequately powered trials using standardized and clinically meaningful endpoints are needed to confirm the reproducibility and clinical relevance of these findings.

## 6. Future Perspectives and Research Roadmap

### 6.1. qPCR-Based Response Monitoring and Prospective Validation

A practical challenge in prebiotic interventions is determining whether an individual is responding and whether the selected prebiotic component, dose, and duration are appropriate. Responses are unlikely to be uniform because baseline gut microbiota composition and functional capacity influence how a prebiotic substrate is utilized. Large human studies showing substantial interindividual variation in response to standardized foods support the broader principle that a fixed nutritional intervention may not produce the same effect in every individual [40,41]. Objective and repeatable measurements are therefore needed to determine whether the intended microbiota response has occurred and whether the intervention should be maintained or adjusted.

16S rRNA sequencing, shotgun metagenomics, and metabolomic profiling can provide detailed information on microbiota composition and function. However, their cost, analytical complexity, and bioinformatic requirements limit their use for repeated monitoring in routine clinical or nutritional settings. Targeted stool DNA qPCR offers a simpler approach for repeatedly measuring a predefined set of microbial gene markers before and during supplementation.

Microbial markers have been combined into classification models for disease-associated gut microbiota patterns, particularly in colorectal neoplasia [42,43]. The same principle can be applied to targeted qPCR by integrating predefined markers into a composite score. At baseline, such a score can characterize an individual’s starting microbial profile; repeated measurements can then be used to track changes after a microbiota-targeted intervention. The pancreatic cancer (PC) probability score described below provides an example of this approach [44].

**Figure 3:**
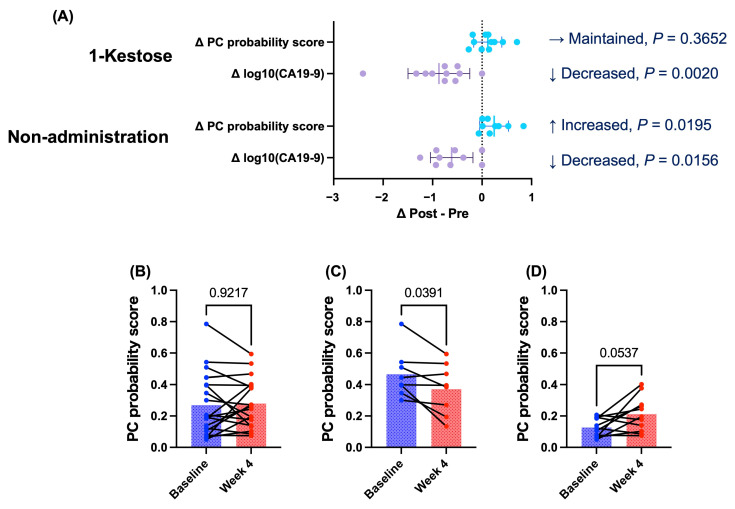
qPCR-based monitoring in two intervention settings. (**A**) Redrawn individual Δ (post−pre) plot summarizing the paired pre/post pancreatic cancer (PC) monitoring analysis reported in [44]. Each point represents the change in PC probability score or log10(CA19-9) in the partial response (PR) subset (1-kestose, *n* = 11; non-administration, *n* = 9). Light-blue symbols and error bars indicate changes in the PC probability score, whereas purple symbols and error bars indicate changes in log10(CA19-9). Summary data are presented as the mean ± SD. The vertical dashed line indicates no change from baseline (Δ = 0). Within-group pre/post differences were assessed using two-sided Wilcoxon matched-pairs signed-rank tests. Panel A uses the same underlying analysis but does not reproduce the original graphical panel from [44]. (**B**–**D**) Post hoc exploratory reanalysis of previously collected healthy-adult data from the 1-kestose plus inulin trial [36], applying the externally defined PC-model cut-off from [44] without re-optimizing the threshold ((**B**), *n* = 19; (**C**), *n* = 8; (**D**), *n* = 11). The overall change was not significant (**B**), participants above the cut-off showed a significant decrease (**C**), whereas those below the cut-off showed a numerical increase that did not reach statistical significance (**D**). Paired baseline and week-4 values were compared using two-sided Wilcoxon matched-pairs signed-rank tests. Blue and red symbols and bars indicate baseline and week 4, respectively, and black lines connect paired observations from the same participant. Numbers shown above the comparisons indicate two-sided, unadjusted *P*-values. Bars indicate the mean. Together, these panels illustrate exploratory qPCR score monitoring in patients with pancreatic cancer and in healthy adults. PC, pancreatic cancer; PR, partial response.

qPCR-based monitoring was explored in two previously conducted intervention studies. First, the PC probability score, a three-marker qPCR score developed in patients with pancreatic cancer [44], was applied longitudinally to residual fecal DNA from a randomized pilot trial of 1-kestose supplementation during chemotherapy [34]. The analysis included only patients who achieved a partial response (PR), defined according to RECIST version 1.1 as at least a 30% decrease from baseline in the sum of diameters of target lesions [34]. Although serum levels of CA19-9, a blood-based tumor marker commonly used to monitor pancreatic cancer, decreased in both groups, the PC probability score increased in the control group but remained stable in the 1-kestose group (Figure 3A) [44]. Because this was a post hoc analysis of a selected subgroup, the result requires prospective validation in an independent pancreatic cancer cohort.

Second, the same score was applied post hoc to data from healthy adults who received 1-kestose plus long-chain inulin [36]. The predefined cut-off of 0.2818, originally derived from patients with pancreatic cancer, was used without modification. No significant change was observed in the full study population (Figure 3B). However, the score significantly decreased among participants whose baseline values were above the cut-off, whereas no statistically significant change was observed among those below the cut-off (Figure 3C,D). These results indicate only a change in a microbiome-derived score and do not demonstrate reduced pancreatic cancer risk or validate the score for screening healthy individuals. Nevertheless, the baseline-dependent pattern suggests that qPCR-derived scores may have potential for estimating the likelihood of a microbiota response to 1-kestose-based interventions and for monitoring that response during supplementation. Prospective studies are needed to determine whether baseline scores can reproducibly identify likely responders and whether changes in the scores are clinically meaningful.

**Figure 4:**
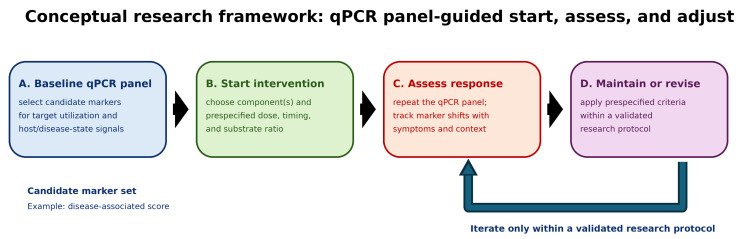
Investigational qPCR-guided workflow for baseline assessment, response monitoring, and prospective adjustment of prebiotic interventions. (**A**) A predefined stool qPCR panel is measured before supplementation to characterize selected microbial markers. (**B**) The intervention is initiated using a prespecified component, dose, timing, and substrate ratio. (**C**) The same markers are measured during or after supplementation and interpreted together with symptoms, gastrointestinal tolerability, diet, and clinical context. (**D**) Future comparative trials can test whether prespecified criteria for maintaining or modifying the intervention improve outcomes relative to a fixed regimen. This conceptual workflow requires prospective validation before clinical implementation.

Building on these exploratory observations, Figure 4 presents a proposed research framework in which a predefined stool qPCR panel is measured at baseline and repeated during or after supplementation. Changes in the selected microbial markers would be interpreted together with symptoms, gastrointestinal tolerability, diet, and relevant clinical outcomes. The panel should be tailored to the intervention goal and target population and may include markers related to the capacity for 1-kestose utilization, responses of target microbial groups, or disease-associated microbiota patterns. Future comparative trials should test whether adjusting the prebiotic composition, dose, timing, or 1-kestose-to-inulin ratio according to prespecified qPCR and clinical criteria improves outcomes compared with a fixed regimen.

Taken together, Figure 3 provides exploratory examples of qPCR-based response monitoring, whereas Figure 4 presents the corresponding framework for prospective evaluation. Prospective comparative trials are needed to determine whether this approach improves response assessment and intervention design relative to a fixed regimen.

### 6.2. Potential Application via the Gut–Brain Axis

The gut–brain axis describes communication between the gut microbiota and the central nervous system and may influence brain function and behavior [45]. In a social-isolation stress mouse model, 1-kestose intake attenuated depression- and anxiety-like behaviors and was accompanied by changes in the gut microbiota, increased SCFA levels, and reduced markers of microglial activation [46]. In dogs with owner-reported aggression-related behaviors, 1-kestose administration was also associated with changes in behavior and gut microbiota composition [15]. Although not specific to 1-kestose, a randomized trial in older adults reported that an inulin–fructooligosaccharide supplement altered the gut microbiota and improved a composite cognitive score relative to placebo [47]. Together, these findings support further investigation of prebiotic modulation of the gut–brain axis, while evidence specific to 1-kestose remains limited to animal studies.

Potential mechanisms include SCFA-mediated modulation of neuroinflammation, immune signaling, vagal pathways, gut-peptide secretion, and changes in tryptophan or bile acid metabolism. Controlled human studies are needed to determine whether 1-kestose produces clinically meaningful effects on mood or behavior and to identify the mechanisms involved.

### 6.3. Applications in Aquaculture and Other Animal Health Contexts

Fructooligosaccharide-based synbiotic strategies have been evaluated in aquaculture, with reported effects on immune function and disease resistance in fish [48]. Within this broader context, 1-kestose-based interventions have also been investigated in diverse animal species, suggesting potential applications in animal health. In Japanese eel aquaculture, co-administration of 1-kestose with heat-killed *Lactiplantibacillus plantarum* FM8 improved feed efficiency by approximately 20% and reduced the abundance of *Edwardsiella*, a genus that includes important fish pathogens [49]. Related applications of 1-kestose-based microbiota modulation have also been explored in Magellanic penguins and captive belugas and were collectively summarized in a conference presentation [50]. These observations support further species-specific evaluation of 1-kestose in animal-health settings.

Because these studies involved different species, interventions, and outcome measures, their findings should be interpreted within each animal-health context. Future studies should assess reproducibility, safety, and species-specific dose and duration, and, where applicable, optimize the co-administered microbial preparation. Repeated non-invasive monitoring of microbiota composition, functional markers, metabolites, and host outcomes may help clarify the mechanisms and practical relevance of these interventions and provide comparative insight into microbiota-mediated effects.

## 7. Conclusions

1-Kestose is a chemically defined, high-purity, low-DP fructan that is generally fermented rapidly by gut microorganisms, particularly in the proximal colon. Its selective microbial utilization is largely shaped by the substrate specificity of GH32 enzymes. The availability of high-purity 1-kestose enables clearer investigation of the relationships among molecular structure, microbial utilization, and host-relevant responses than mixed FOS preparations. In contrast, long-chain inulin is fermented more gradually, providing a mechanistic rationale for testing the two fructans in combination.

Human intervention studies have reported potentially beneficial effects of 1-kestose across several clinical settings, although many remain small-scale or exploratory. Larger independent randomized trials are needed to confirm efficacy, safety, and tolerability. Direct comparisons of 1-kestose alone, long-chain inulin alone, and their combination will also be required to determine whether co-administration provides additional benefits.

The integration of high-purity 1-kestose, GH32-dependent microbial selectivity, and qPCR-based baseline stratification and response monitoring provides the basis for evaluating 1-kestose as a candidate precision bioactive component. In this framework, baseline microbial markers could help estimate the likelihood of a microbiota response, and repeated qPCR measurements could be used to monitor changes during supplementation. Prospective trials should determine whether these measurements can guide the selection of dose, timing, or co-administration with long-chain inulin and improve the reproducibility and clinical relevance of prebiotic interventions.

## Figures and Tables

**Table 1:** Summary of representative human intervention studies of 1-kestose.

Population/Condition	Intervention	Key Outcomes (Selected)	Ref.
Ulcerative colitis (UC) /Patients with mild–moderate UC	1-kestose 10 g/day, 8 weeks; randomized, double-blind, placebo-controlled pilot trial. *N* = 40; *n* = 20/20.	Improved clinical remission rate; reduced Lichtiger score; decreased *Ruminococcus gnavus* abundance.	[28]
Crohn’s disease (CD) /Patients with CD in remission	1-kestose 3 g/day, 12 weeks; single-arm pilot study. *N* = 19.	Remission symptoms remained stable; reduced fecal inflammatory marker levels.	[29]
Insulin resistance/Prediabetes Overweight/obesity-prone adults	1-kestose 10 g/day, 12 weeks; randomized, double-blind, placebo-controlled trial. *N* = 38; *n* = 20/18.	Lower fasting insulin; improved HOMA-IR; increased *Bifidobacterium* abundance.	[30]
Atopic dermatitis /Infants	1-kestose 1–2 g/day, 12 weeks; randomized, double-blind, placebo-controlled trial. *N* = 65; *n* = 33/32.	Improved SCORAD score; promoted early establishment of *Faecalibacterium prausnitzii.*	[31]
Cow’s milk allergy /Children	1-kestose 2–4 g/day, 24 weeks; open-label parallel-group comparison. *N* = 22; *n* = 13/9.	Decreased serum IgE; inverse correlation between *Fusicatenibacter* abundance and IgE levels.	[32]
Low birth weight neonates /NICU-admitted premature/low-birth-weight neonates	1-kestose 0.3 g/day, 4 weeks; randomized comparison with lactulose in NICU neonates receiving *Bifidobacterium breve*. *N* = 26; *n* = 13/13.	Increased *Bifidobacterium*; reduced *Staphylococcus aureus*.	[33]
Pancreatic cancer (during chemotherapy) /Patients receiving chemotherapy	1-kestose 9 g/day, 12 weeks; single-center randomized controlled pilot trial. *N* = 38; *n* = 20/18.	Reduced CA19-9; preserved nutritional status (albumin); shifted gut microbiota (including reduced *Escherichia coli*).	[34]
Constipation regulation /Children (kindergarten age) with constipation	1-kestose 3 g/day, 8 weeks; double-blind, randomized, placebo-controlled pilot trial. *N* = 23; *n* = 11/12.	Significant increase in bowel movement frequency; improved stool form.	[35]
Healthy adults /Adults	1-kestose 3 g plus long-chain inulin 3 g/day, 4 weeks; randomized, double-blind, placebo-controlled trial. *N* = 37; *n* = 18/19.	Increased *Bifidobacterium* abundance.	[36]

Note: *N* is the total study sample; *n* gives group sizes in the order stated in the intervention column. For single-arm studies, only *N* is shown. The healthy-adult combined-fructan trial compared the combination with placebo and did not include component-only groups.

## Data Availability

No new datasets were generated through new participant recruitment or new sample collection for this review. The post hoc exploratory reanalysis shown in Figure 3 used previously collected data and/or residual fecal DNA from the source studies [34,36,44]. Data underlying these analyses are available from the corresponding author upon reasonable request, subject to institutional and ethical restrictions.

## Supplementary Materials

The following supporting information can be downloaded at: https://www.mdpi.com/article/10.3390/foods15152631/s1, Supplementary Table S1. GH32 Proteins Shown in Figure 2. Abbreviated labels used in Figure 2 and the corresponding organism, strain, protein designation, accession number, phylogenetic cluster, evidence status, and supporting reference.

## Abbreviations

AD, atopic dermatitis; CD, Crohn’s disease; DP, degree of polymerization; FOS, fructooligosaccharides; GF2, 1-kestose; GH32, glycoside hydrolase family 32; IBD, inflammatory bowel disease; NICU, neonatal intensive care unit; PC, pancreatic cancer; PDAC, pancreatic ductal adenocarcinoma; qPCR, quantitative polymerase chain reaction; SCFA, short-chain fatty acid; SCORAD, Severity Scoring of Atopic Dermatitis.
