# Supplementary material for: 1-Kestose as a Candidate Precision Bioactive Component: From GH32-Dependent Gut Microbiota Regulation to Human Health Enhancement
Source: Foods. 2026 Jul 27;15(15):2631. doi: 10.3390/foods15152631 (PMC13464611; doi:10.3390/foods15152631)
Supplement: Supplementary file 1 [file foods-15-02631-s001.zip › foods-4411211-supplementary.pdf]

Supplementary Table S1. GH32 Proteins Shown in Figure 2

Abbreviated labels used in Figure 2 and the corresponding organism, strain, protein designation, accession number, phylogenetic cluster, evidence status, and supporting reference.

Figure label	Organism / strain	Protein designation	Accession number	Cluster	Status / context	Ref.
BL-CscA	*Bifidobacterium longum*	CscA	KFI62304.1	1	Reference GH32 protein	[13]
BC-GH32	*Blautia caecimuris*	GH32	MCR2002371.1	1	Biochemically characterized	[15]
RIV-GH32	*Roseburia inulinivorans*	GH32	ADF57367.1	1	Reference GH32 protein	[13]
AR-GH32-1	*Agathobacter rectalis*	GH32-1	ACR74061.1	1	Reference GH32 protein	[13]
RF-GH32	*Roseburia faecis*	GH32	MFQ9042080.1	1	Reference GH32 protein	[13]
CE-GH32-1	*Coprococcus eutactus*	GH32-1	GFO93478.1	1	Reference GH32 protein	[13]
CE-GH32-2	*Coprococcus eutactus*	GH32-2	GFO94608.1	1	Reference GH32 protein	[13]
PD-GH32	*Parabacteroides distasonis*	GH32	BEJ45010.1	3	Biochemically characterized	[14]
RIN-GH32	*Roseburia intestinalis*	GH32	EEV01593.1	3	Reference GH32 protein	[13]
PG-GH32	*Parabacteroides goldsteinii*	GH32	BEJ45011.1	3	Biochemically characterized	[14]
AH-GH32-4	*Anaerostipes hadrus*	GH32-4	EKY22990.1	3	Reference GH32 protein	[13]
AB-GH32-3	*Anaerostipes butyraticus*	GH32-3	GFO85652.1	3	Reference GH32 protein	[13]
FD-GH32	*Faecalibacterium duncaniae*	GH32	ATP01188.1	3	Reference GH32 protein	[13]
AC-S6PH	*Anaerostipes caccae*	S6PH	EDR98961.1	2	Reference sucrose-oriented GH32 protein	[13]
AB-GH32-1	*Anaerostipes butyraticus*	GH32-1	GFO84605.1	2	Reference GH32 protein	[13]
AH-GH32-1	*Anaerostipes hadrus*	GH32-1	EKY24952.1	2	Reference GH32 protein	[13]
AH-GH32-2	*Anaerostipes hadrus*	GH32-2	EKY21880.1	2	Reference GH32 protein	[13]
AB-GH32-2	*Anaerostipes butyraticus*	GH32-2	GFO83738.1	2	Reference GH32 protein	[13]
FN-GH32	*Fusobacterium nucleatum* CP11	Predicted GH32	BEO99072.1	2	Sequence-based prediction; pathogen-associated strain (Cancer-associated oral-gut bacterium linked to colorectal cancer tissue colonization and oral-gut transmission.)	[19]
SA-GH32	*Staphylococcus aureus* Mu50	Predicted GH32	BAB58203.1	2	Sequence-based prediction; pathogen-associated strain (Vancomycin-intermediate *S. aureus* (VISA) strain; antimicrobial-resistant pathogen)	[18]
SANG-GH32	*Streptococcus anginosus* NCTC10713	Predicted GH32	VED98926.1	2	Sequence-based prediction; potentially pro-inflammatory strain (Abscess-associated and potentially pro-inflammatory oral *Streptococcus* lineage; used as a pathogenic/reference strain context)	[20]
CP-GH32	*Clostridium perfringens* MFDS1012647	Predicted GH32	UYC91955.1	2	Sequence-based prediction; pathogen-associated strain (Enterotoxigenic, food-poisoning-associated strain isolated in the context of a restaurant outbreak; pathogenic/toxin-related genes were reported in the strain description)	[16]
CD-GH32	*Clostridioides difficile* R20291	Predicted GH32	CBE04456.1	2	Sequence-based prediction; pathogen-associated strain (Epidemic 027 outbreak strain; representative toxin-producing nosocomial pathogen.)	[17]
CB-GH32	*Clostridium butyricum* CBM588	Predicted GH32	WLS68050.1	2	Sequence-based prediction; human probiotic strain (included as a clostridial comparator rather than a pathogen)	[21]

Note: Cluster assignments are based on amino acid sequence-based phylogenetic analysis. For predicted proteins, cluster placement does not establish substrate specificity or whole-cell utilization of 1-kestose. Reference numbers correspond to the revised manuscript.
